# Understanding the Current State of Play of Early Intervention for Bipolar Disorder: Qualitative Analysis of Consultations With International Stakeholders

**DOI:** 10.1111/bdi.70091

**Published:** 2026-02-16

**Authors:** Sue M. Cotton, Vani Jain, Aswin Ratheesh, Clare Shelton, Craig Macneil, Kate M. Filia, Jacob Crouse, Annabel Burnside, Emily Clarke, Alesha Prasad, Rosie Arnold, Paul Badcock, Melissa Hasty

**Affiliations:** ^1^ School of Psychological Sciences Monash University Clayton Victoria Australia; ^2^ Turner Institute for Brain and Mental Health Clayton Victoria Australia; ^3^ Centre for Youth Mental Health University of Melbourne Parkville Victoria Australia; ^4^ Orygen, the National Centre for Excellence in Youth Mental Health Parkville Victoria Australia; ^5^ Daymark Foundation Toronto Ontario Canada; ^6^ Brain and Mind Centre University of Sydney Camperdown New South Wales Australia

**Keywords:** at‐risk, big data, bipolar disorder, community awareness, early intervention, education, families, outcome measurement, service models, treatment

## Abstract

**Background:**

Despite the burden associated with bipolar disorder (BD), research into early diagnosis and treatment of BD lags approximately 20 years behind the field of early intervention for psychosis. This study evolved through a partnership between Orygen (Melbourne, Australia) and the Daymark Foundation (Toronto, Canada). The primary focus was to answer the question: “How might we advance an early intervention approach for people at‐risk of or with BD?”.

**Methods:**

Semi‐structured interviews were conducted with international experts and other stakeholders in early intervention and BD to identify challenges and barriers in early intervention approaches to BD.

**Results:**

Twenty‐eight experts participated. Nine themes emerged as challenges: (i) limited recognition and understanding of BD across stakeholders; (ii) lack of definitions; (iii) poor resourcing and lack of prioritisation in funding models; (iv) absence of validated tools for diagnosis, monitoring treatment response and/or disorder progression; (v) absence of “big data”; (vi) scarcity of evidence‐based treatments and clinical guidelines for the early stages of the disorder; (vii) obscurity around optimal service models; (viii) the need for better support and involvement of families and significant others; and (ix) the need for collaboration (across disciplines, stakeholders, and settings) to progress the field.

**Limitations:**

Of those approached, 54.9% participated in the study. Given the qualitative nature of the study, recruiting more experts to the study would not necessarily change the outcomes as data saturation was achieved.

**Conclusions:**

This work lays the foundations for developing a collaborative research framework to progress early intervention for young people with BD.

## 1 Introduction

Bipolar disorder (BD) is an enduring and severe mental health disorder that commonly has its onset before the age of 25 years [1, 2]. Over the disorder's course, individuals can experience depressive and subsyndromal affective symptoms for ~50% of their lives [3]. Despite recovery, functional impairments persist in social [4], occupational [5, 6, 7], psychological and environmental domains [8]. Early mortality can be due to suicide (rates as high as 20%) [9] and preventable physical comorbidities (e.g., cardiovascular disease) [10, 11]. BD is associated with marked burden particularly for the 15–24 years age group [12].

The pathophysiology of the BD is largely unknown [13, 14], making it difficult to identify those at risk of developing the disorder [14]. Diagnostic imprecision is common and there can be years between symptom onset and diagnosis [1, 15]. During this period, individuals may receive inappropriate, ineffective, and/or iatrogenic treatments [16].

Treatment innovation is lagging for BD [17]. Lithium, the mainstay treatment for BD, was discovered in 1949 [18] and most other pharmacotherapies were developed for other disorders (e.g., antipsychotics). Because of treatment ineffectiveness polypharmacy is the norm [19] and can be associated with marked side‐effects [20]. Clinical guidelines highlight the benefits of psychotherapy [21]; however, these therapies are not easily accessed, clinicians lack experience in their delivery, and they are costly [22, 23]. Some data suggest if effective and less aggressive treatments are provided in the early stages of the disorder, then the long‐term burden might be lessened [24, 25, 26]. Research is scant, however, on interventions for the early stages of BD [24, 25, 26, 27].

The potential burden of BD underpins the urgent need for prevention, early identification, and treatment access for BD [28]. Despite this need, research on early intervention for BD lags decades behind the field of early intervention for psychosis (EIP) [14, 24]. Although there is agreement that we should support young people at‐risk and during onset or the early stages of BD [1, 24, 25, 29], it is unclear how best to advance this work, and many unanswered questions remain.

This qualitative study was driven by a partnership between Orygen (world leader in early intervention, Australia) and the Daymark Foundation (private family foundation with a focus on BD, Canada). We asked the question: “How might we advance an early intervention approach for people at‐risk of, or with, BD?”. Early intervention was conceptualised as “diagnosis at the earliest possible point, even pre‐symptomatically, followed by proportional or stage‐specific intervention adapted and sustained for as long as is necessary and effective” (p. 545) [30].

## 2 Materials and Methods

### 2.1 Design

Semi‐structured interviews with experts and stakeholders in the field of early intervention for BD were conducted. Reporting of the study's findings was in accordance with the Standards for Reporting Qualitative Research (SRQR) [31].

### 2.2 Researcher Characteristics and Reflexivity

Four senior team members (S.M.C., M.H., C.M., C.S.), each with 20+ years in youth mental health, conducted the interviews. Three were trained in clinical psychology (M.H., C.M., C.S.) and the other in neuropsychology (S.M.C.).

### 2.3 Sample

The target population was international experts who had worked with young people or adults at risk of, or experiencing, the early stages of BD in clinical and/or research settings. Other included non‐government and philanthropic organisations who support BD research. Experts were identified using a multi‐pronged approach: (i) the research team's own familiarity with the field and established networks; (ii) a scoping review of recent literature to locate authors publishing in early intervention; (iii) consultation with relevant professional bodies; and (iv) snowballing techniques to identify further candidates.

### 2.4 Interview Schedule

Because “early intervention” can have different meanings, we provided this preamble: “While the term “early intervention in BD is sometimes used to refer only to treatment provided at the first manic or hypomanic episode, for these questions we are using it in the broader sense of also supporting individuals displaying early but significant mood symptoms (which may or may not be diagnosed as BD), as well as preventative interventions for individuals with major risk factors for developing BD”. The scope of interventions was also set: “… the term “intervention” does not necessarily mean pharmacological treatment and can include any other form of care and support to an individual and/or their family (e.g., psychosocial)”.

The semi‐structured interview comprised nine questions covering: (i) research gaps, research challenges, and identifying what works; (ii) challenges with interventions for at‐risk and early stages of BD; (iii) problems with the early detection of BD; and (iv) challenges faced by the field more broadly (see Table 1 for questions).

**TABLE 1:** The interview schedule for Phase 1 of the study.

For the first part of this interview, we have some broad questions regarding *how we might advance BD early intervention research*
What are the major research gaps related to early intervention in BD?
2 What are the challenges in conducting research on early intervention in BD?
3 Given our current state of knowledge, what evidence could be translated to practice now and how could we do it?
For the next part of this interview, we want to talk about *interventions*. A range of biological and psychosocial treatments (including digital interventions) are available for BD
4 What do you think are the biggest challenges in implementing any of these interventions in the early post onset stage? (By “*early post onset stage*” we mean intervention with young people who have had their first significant mood episode, or are within the first few episodes)
5 With respect to any of these treatments, what do you think are the biggest gaps or challenges in implementing preventive interventions for young people at = risk of BD? (By “*Preventive intervention*” we are referring to intervention for persons at a higher risk of developing BD. “*Young people at‐risk of BD*” may include persons with a family history of BD, or those at higher risk due to experiencing depressive symptoms and or hypomanic symptoms, but not actually developed enough symptoms as to be diagnosed with BD)
Next, we want to talk about *early detection*
6 What are your personal “best bets” on how we can reduce the delay in diagnoses and early treatment for BD?
7 What are the most important steps to improving prediction of BD? (By “*prediction*” we mean identifying who might be at high risk of developing BD. For example, these may include young people with a family history of BD, or those at higher risk due to experiencing depressive symptoms and or hypomanic symptoms)
Finally, we want to talk about *the field*
8 What characteristics of the early intervention and/or BD space make it challenging to advance an early intervention approach in BD?
9 What are some promising examples or opportunities for advancing a common agenda for early intervention in BD?
Is there anything else you want to share with us about the challenges, barriers, or opportunities related to advancing an early intervention approach in BD?

### 2.5 Procedure

Ethics approval was obtained from the University of Melbourne (#26552). Experts were sent an introductory email describing the study. For participants, a 1 h Zoom meeting was scheduled where verbal consent was obtained, and the interview conducted. The interview was recorded, and a transcription document was automatically generated. Research assistants checked the quality of the transcription against the audio recordings, and the transcription was amended as required. Interviews occurred between May and June 2023. Participants received a CAD $200 honorarium (Visa gift card).

### 2.6 Analyses

Transcripts were examined using reflexive thematic analysis [32]. This framework comprises six steps: (i) familiarisation with the data; (ii) developing code labels capturing meaning from data segments; (iii) generating initial themes from the coded data; (iv) reviewing the accuracy of initial themes; (v) refining, defining, and labelling themes; and (vi) report generation [32]. Analyses were led by SC and MH. Each independently reviewed transcripts and labelled meaningful segments without having preconceived themes. They met and discussed initial themes, resolved any disagreements through consensus, and fine‐tuned the final themes.

## 3 Results

Fifty‐one experts were approached and 28 consented to partake across 27 interviews (two participants were interviewed together). Participants included clinical researchers (*n* = 19), researchers (*n* = 3), clinicians, peer support workers (*n* = 2), philanthropic funders, and representatives from BD advocacy organisations (*n* = 4). Some participants disclosed a lived experience of BD (personal experience or as a caregiver). Those who were interviewed were from the US (*n* = 8), Canada (*n* = 8), Australia (*n* = 4), UK (*n* = 4), Europe (*n* = 3), and one was from South America. Of the 23 who were contacted but did not response or declined to participate most were clinical researchers (*n* = 17), followed by researchers (*n* = 4), and support or philanthropic organisations (*n* = 2). They had similar geographic locations to those who participated; most were from US (*n* = 8), Canada (*n* = 6), Australia (*n* = 5), UK (*n* = 3), and one was from South America. Because of the small sample sizes, especially across subgroups (i.e., whether or not participated in the interviews, expert types and geographic location), we did not undertake direct examination of differences.

From the interviews, nine themes were identified (see exemplar quotes in Table 2).

Theme 1 *Awareness*. “*There is limited recognition and understanding of BD (including early warning signs and at‐risk states) across all stakeholder groups, from the general community through to mental health providers, clinicians, and services*”.

**TABLE 2:** Nine themes identified from the semi‐structured interviews pertaining to insights on challenges, barriers and opportunities.

Theme	Quote
Awareness	“So I mean, I think in the end it will be going to the public, and saying, “You know, if you have, if there is bipolar disorder in your family, then these may be the things that you need to look out for”, because I don't think our services, I don't think they're going to do it… to actually go direct to the community and say, these are the things that you might want to look out for, and these are some good screening tools for early bipolar disorder, for severe mood disorders, and come to a clinic with that information. So can go direct to consumers is one way of doing it. I think more education with youth services around the country…” [13] “I mean even people who I think of as being really, you know, good, competent, clinical psychiatrists often miss low grade manic symptoms… And I have colleagues who treat young people with mood disorders, and who, you know, prescribe someone an antidepressant, and really don't recognise when the kids getting really kind of revved up and irritable that there are some mixed symptoms emerging. It's, yeah, the mixed states really do seem to go unrecognised a lot, even by people who are otherwise clinically quite good [6].
Definitions	“… We have confusion about what is bipolar in young people. At what age can it start? Can it be Childhood? 75% fold difference between the UK and America in diagnostic rates of bipolar in children. You know it's the least accurate or the least reliable internationally… Building international consensus around what you're trying to do is quite difficult, because it's not even clear people are talking about the same sorts of populations… The first thing is, we have to agree some definitions in bipolar…” [9]
Resourcing	“Yeah, well look bipolar disorder's the most neglected of the top 10 causes of disability in terms of research funding, prioritisation, novel therapy development, you name it. We're at the bottom of the pile, and that's crazy given that it's the one disorder we really can make a difference at the stage with what we know, but we don't do it. So I think the biggest problem is systems based… So you have to have a health care system that is resourced and empowered and enabled to deliver evidence based care. Now that's not available in most settings” [2]
Measurement and Assessment	“We have no objective measures. It's hard to define what you got in front of you right, because you can send the same patient, to 6 different psychiatrists, for example, we can get oh 6, if not 12, different diagnoses you know, even if you use a structured clinical interview. It's not clear if you follow those people over time. I mean not to mention the fact that DSM diagnosis doesn't necessarily map on to the treatment right?… because we have no objective measures of this thing clinical practice is varied, quite widely from site to site… and you know, to be honest, I think one of the biggest obstacles is people don't follow patients. I mean, we don't. We don't have health systems that do a particularly good job following patients over time, and you cannot detect BD if you're just taking a snapshot. You know you couldn't unless, until they're really, really severely ill. The hospitalised, psychotic patient, that's pretty clear. Yeah, but if you're talking about early detection. They need to be watched over time. They need to be monitored over time” [5]
Treatments	“I think, quite a dearth of comparative effectiveness data, such that we cannot really match these young patients to treatments. Yeah. And what we would really like to have is some sense of can you in any way predict what's going to be the best treatment for any given individual? And I think a mixture of personalised and precision treatment for these people is really needed. And of course, like you'll hear from many people, we don't have biomarkers right. There's nothing biological that may at this point help, and I think we need to explore that. To try to see is there anything we can understand about biology that will lead to a better matching of the pharmacologic treatment and the psychosocial treatments” [10] “I think there's a bunch of challenges that the one is a lack of a real robust evidence space in early stage patients to know which treatments choose first, how to sequence them, what doses of medications to prescribe, which psychological interventions are the best. All the usual acceptability problems, you know, stigma and denial, adherence problems in the young people we're treating. You know, I think there's even in treated samples, like I worked in Lakshmi's first episode of mania program for 10 years before I moved to the US, and, you know, we did our best to provide treatment for these young people with medications and other interventions, and the relapse rates were still pretty high. 40% over a year, I think, 60%–65% over 3 or 4 years. What we have is not enough. And I think that really speaks to the importance of team based interventions and the coordinated specialty care sort of model of treating the whole patient and not just prescribing them with treatment. But right now our treatments are just not enough, and so it's really kind of hard for us to make a really strong case to people that you need to stay on these medications when the relapse rates are so high, especially given that the side effects are also pretty bad for a lot of medications for sure. Weight gain, metabolic problems, you know, motor dysfunction, all kinds of pretty not nice stuff. So I feel like the lack of an evidence base, the lack of really robustly effective treatments, and the really high side effect burden create a lot of challenges for implementing, again I'm a prescriber, so I tend to think more about medication treatments, but make it hard to implement some of our best interventions” [6].
Service Capacity and Models	“I suppose you know a lot of the feedback that I get from our members is that the reason they felt they didn't get a diagnosis earlier is that they weren't actually listened to by health professionals, and also that here, most people will present to their GP, their family doctor, the general practitioner, you know, when they're first experiencing symptoms, and there just seems to be a lack of understanding, of knowledge of bipolar, of things like psychosis, of understanding that hypomanic episode might look like for them to be able to identify it's not depression, you know. There's more going on here” [19] “A lot of clinicians see it as a variant of schizophrenia which needs roughly similar treatment, and it absolutely is not so we have that challenge… Well that's the other problem with BD, there can be very few disorders in the whole of psychiatry, where current clinical practice is further removed from the evidence base. So if you look at what the evidence base says, how you should treat the disorder and what actually happens out in practice, almost nobody gets evidence based therapy” [2] “There doesn't seem to be a recognition of mixed states, or dysphoric mania, dysphoric hypomania. it's this narrow mindedness about this thing… I've actually had residents say to me, oh, I can't possibly diagnose someone with bipolar even though they the patient, clearly had a manic episode I'm like well, why? Not well, because that's a lifetime and serious mental illness. No, no, it's like, okay but do you want the next person to see this person to slam them with antidepressants, because we know that makes them worse. Isn't that really a good outcome” [5] “So I think it's a fear of the diagnosis and a fear of the medication. I'm. Actually surprised how few of the psychiatrists now use any of the classical mood stabilisers as well. It's as if they're afraid of the medication. And they are afraid of the diagnosis… and it's also peculiar like you'd rather label this person as treatment refractory depressed” [5]
Families	“Well, I think that you know the emphasis in intervention now is really heavily focused on biological intervention. So, achieving some better balance there, looking at more systematic family involvement, and better psychoeducation, those would be key things… especially when you're talking about young people, there is family involvement, for which we have good evidence… it needs to be much more standardised and brought into practice” [7] “So you know, we certainly have that point of view that if someone's support network, if they are informed, if they are well equipped, if they have support, then that's ultimately going to benefit the person who is struggling with bipolar” [19] “… family psycho education, monitoring of how the kids are doing, and getting some support and family therapy in there to help prevent the kids from going off the rail in terms of more severe depression, that the family therapy things are the areas where we have the best control data actually… But then, getting kids into that kind of treatment and families into that kind of treatment. It is very difficult” [16]
Collaboration	“It feels to me like what we're going to need are kind of moon shots, right? The kind of engineering that got us to the moon where you really get a lot of the engineers together, and you fly a pretty big rocket. You need large enough sample sizes with experts who have a lot of different models that they're interested in. And so then I think what happens is that there is base… And so I see the biggest gap, and the reason I was excited to see how much you guys are doing reach out, is bringing people together to work very, very collectively on the solutions… I think it's really important to get a lot of people together but also not just to ask about the things we're proud of but the things that people really want to change” [21]
Data	“I mean, big data is one… getting people across countries together with the idea of agreeing on what the common challenges are, working together on what the common solutions are, agreeing to common measures. Can you imagine across countries? If we had common measures, and we could actually pool our data and look at things in a really big way? It would be huge” [7]

In the community, trivialisation and casual dilution of terminology [Participant ID: 7, 14], stigma [3, 12, 15, 18, 24, 26], and denial [3, 12, 15, 18, 24, 26] were viewed as barriers to help‐seeking, diagnosis, acceptance, and engagement in treatment and research. Two participants reported that denial is common in family members and young people, resulting in attribution of symptoms to alternate causes (e.g., adolescent mood fluctuations) [20, 26]. Education to improve recognition, timely diagnosis, and treatment was considered necessary for the general community [3, 9, 13, 14, 19, 20, 24], families [7, 11, 13, 15, 16, 19, 20, 24–26], schools [3, 20, 26] and peers [26].

Participants were clear that general practitioners [11, 15, 18, 19, 21], primary health care practitioners [8, 9, 16, 18, 19], paediatricians [10, 26], and mental health clinicians [3, 8, 10–16, 19, 20, 23–26] need education regarding screening for BD [3, 6, 8, 13, 15, 20] including for family history of BD [3, 7, 9–11, 13, 14, 16, 19, 20]. Two participants reported that clinicians can be reluctant in diagnosing BD [5, 15] and others indicated that diagnoses such as depression, psychosis, and/or borderline personality disorder (BPD) are more likely to be made than BD [3, 5, 8, 9, 11, 13, 14].

Theme 2 *Definitions*. “*There is a lack of consensus on key aspects of early intervention in BD including definitions of at‐risk states and what age groups we should be focusing on*”.

The field was viewed as lacking consensus on: (i) what quantifies/qualifies as an “at‐risk” state [6, 8, 9, 15, 18, 19, 21, 22]; (ii) how to define “diagnostic delay” [9, 18]; (iii) what characterises the “early stage of BD” [8]; (iv) at what “age” can BD be diagnosed [7, 9, 10, 15, 16, 20]; (v) what is “early intervention” [8]; and (vi) what “measurement tools” should be used in clinical and/or research settings [22]. Because of variability in definitions, some believed that cohorts differ internationally [9, 15, 16].

Theme 3 *Resourcing*. “*BD is under‐prioritised and underfunded relative to other mental health disorders especially considering the personal and economic costs associated with the disorder*”.

Highlighted as a significant barrier was the limited prioritisation and funding for BD [2, 5, 6, 9, 13–21]. In clinical settings, funding was considered limited for therapy [2, 9, 15, 17–19] and workforce training [15]. In research, BD was described as at the “bottom of the funding pile” [2, 5, 6, 14, 16, 19, 20]. There was the view that traditional funding bodies were uninterested in BD [6, 16, 21]. Early intervention research funding was considered disproportionately directed to psychosis [6, 19, 20], despite the high potential of early intervention for BD [2, 9]. Identifying alternatives to traditional funders was described as necessary [16]; philanthropic investment was viewed as essential for supporting early research, fostering collaborations, and providing data to support future funding applications [6, 13, 16, 21].

Theme 4 *Measurement and Assessment*. “*There is a lack of a core battery of validated tools and approaches to help diagnose, monitor, and measure outcomes in at‐risk and early‐stage bipolar groups*”.

Measurement was seen as problematic for delineating at‐risk states, diagnosis, and monitoring changes over time [5, 7, 9, 15, 17]. BD was considered the most internationally unreliable diagnosis [9]. Although structured diagnostic interviews were noted to be the best option for diagnosis [15], they were considered too long [15]; clinicians have poor training in their use [15], and they lack diagnostic reliability [5]. Participants also highlighted that diagnosis was complicated by a lack of skill and resources to assist with differential diagnosis [5, 13, 19, 23] and assessment of comorbidities [1, 3, 15, 16, 20].

Because of the heterogeneity of BD, some participants suggested that multiple tools are necessary to assess different phenomena (e.g., (hypo)mania, depression, psychosis) [7, 13]. Existing tools were viewed as inadequate and burdensome [7, 9, 13, 15]. Common measures of depression (e.g., Montgomery Åsberg Depression Rating Scale) were criticised for not detecting atypical depression [2] and for being insensitive to differences between unipolar and bipolar depression [2, 11]. Hypomania and mixed episodes were considered difficult to assess with current tools [8, 9] and are often missed by clinicians [6].

Temporal assessment was viewed as essential for BD [5, 7, 8, 13, 27]. Tools were reported to be inadequate for retrospective mapping of mood episodes (necessary for diagnosis) [7, 8] and for prospective assessments [5, 7, 13]. Some suggested that digital and assistive devices and applications might be useful for temporal monitoring, but this requires further exploration [7, 13, 27].

Several comments were noted about youth assessment. Some participants highlighted that youth rarely present when hypomanic, so BD gets missed [3, 8, 19]. When young people present with depression, participants noted that clinicians fail to consider bipolar depression [3, 20], do not look for signs of atypical depression (e.g., hypersomnia) [3, 8, 20] and/or antidepressant non‐response [6, 8, 13].

Theme 5 *Data*. “*We need more meaningful data, harmonisation of existing data, as well as development of bigger prospective datasets*”.

A range of problems was identified with current research and associated data. The first issue pertains to data relevance. Data collected tends to have little meaning to individuals with BD and their families [26]. Some noted that cultural, ethnic, and gender diversity are not addressed [13, 24]. Others highlighted the need to focus on functional outcomes [13, 26].

Second, there is a scarcity of data. Data from randomised controlled trials were limited, and few trials have focused on early‐stage BD; as a result, there is a limited understanding of what works and why [5, 6, 9, 14, 16, 18, 22].

Third, participants indicated that there are issues with types of data collected. Several highlighted the inadequacy of cross‐sectional data and the need for longitudinal and real‐time data, especially around sleep and functioning [5–7, 13, 14, 16, 18, 23].

A final issue relates to the pooling and harmonisation of data. Participants suggested that we should pool datasets (including family history, multi‐presentation symptom profile, clinical characteristics, omics, biomarkers, lithium response, genetic, brain imaging, electronic medical records and registry data, Ecological Momentary Assessments (EMAs)/digital technologies, etc.), and use advanced analytical methods such as machine learning approaches to advance our understanding of BD [9, 14, 17, 20, 22]. Pooling of data (whether existing or prospective data) was viewed as limited due to the heterogeneity of definitions [9], no consensus on minimum datasets [22], absence of common templates for assessments [14], lack of systems to facilitate data collection across sites [13], difficulties in recruiting participants [12], and the lack of specialist services [18].

Theme 6 *Treatments*. “*The evidence‐base for early intervention treatments in BD is scarce, including which treatments should be offered to which individuals, and when and how treatments should be delivered*”.

Participants mentioned that medication, psychosocial and family interventions, peer‐support, and digital interventions may be useful for early intervention. Some noted that there are no clinical guidelines for at‐risk or early stages of BD [8, 17, 23] and no agreement on the adequate dose of early intervention (e.g., should it be 5 years of optimal care?) [2]. These issues stem from the lack of large randomised controlled trials (RCTs) for the early stages of the disorder [5, 6, 9, 14, 16, 18, 22].

For medications, participants highlighted a lack of robust evidence for use, dose, and sequencing of mood stabilisers, antidepressants, antipsychotics, nutraceuticals, and novel therapies in the early stages of the disorder [6, 10, 27]. Some said that lithium, the gold standard treatment, should be used more frequently [6–8, 15, 16, 23], due to its potential as a “disease modifying” agent if used early [3]. Others spoke about challenges associated with selection of first‐line agents for young people [5, 6], the need for more comparative studies [16], and studies evaluating combinations of medication [14, 16], particularly in the presence of various comorbidities [10, 16].

Antidepressant prescription was raised as a concern. Some suggested that more attention needs to be paid to adverse reactions and/or non‐response to antidepressants [5, 6, 8, 13] due to their potential harm in BD (e.g., triggering mania) [2, 3, 8, 10, 15, 24, 27]. Convincing both people with BD and clinicians that mood stabilisers are preferrable to antidepressants was reported to be challenging [2, 3, 5].

Participants highlighted that more information is required on cost‐benefits of medications [3–6, 8, 10, 15, 26, 27]. In young people, participants noted that there was no information on the appropriate duration of medication prescription [23] and whether their treatment response and side‐effect profiles differ from adults [6, 10]. One participant argued that it is hard to make a case to young people to stay on medication when relapse rates are high and side‐effects significant [6]. Physical health monitoring (metabolic, immune, inflammatory) and intervention, some of which can be predictive and/or preventive, was considered poorly integrated into mental health treatment [7, 13, 17, 27].

The overemphasis on medication as the mainstay treatment for BD was noted [7, 20] and despite international guidelines recommending psychosocial interventions, few people receive them [20]. Two participants described that it is not only difficult for people to access therapy, but clinicians do not have the training, skills, and confidence to deliver BD‐specific psychosocial treatments (e.g., cognitive behaviour therapy (CBT) for BD, Family Focused Therapy (FFT)) [10, 20].

Psychosocial interventions can be a benign treatment for at‐risk populations; however, their evidence‐base was considered limited [6, 22]. Some psychosocial interventions were reported to have been trialled on young people or in the early stages of BD (e.g., FFT) while others have been trialled less (e.g., interpersonal and social rhythm therapy (IPSRT)). There was agreement that more evidence on psychosocial interventions was required [6, 9, 11, 22]. Questions were also raised about active ingredients, targets, or mechanisms of effective psychosocial interventions (e.g., exercise, sleep and circadian and social rhythms, diet, substance use) [10, 20]. The lack of developmentally appropriate treatments for young people was also noted [2, 24].

Peer support interventions were viewed as valuable [11, 17, 19, 24, 26]; providing helping individuals and families to accept diagnosis (11). Whether such interventions are best delivered one‐on‐one or in groups remains unclear. It was suggested that lived experience and family peer support groups need to bring together people with common experience (e.g., age, developmental phase, and stage of disorder of the young person with BD) [26, 27].

Digital interventions were viewed as under‐utilised [13, 15, 17]. Promising areas for use of these technologies included: low intensity interventions [20]; interactive websites [15], tools to facilitate engagement [7]; filling gaps in clinician skill [15]; operationalising CBT initiatives or other psychosocial interventions [7]; interactive games (although these are hard to conceive) [15, 27]; engaging with a clinician remotely [15]; immediate accessibility (wherever, whenever people want) [19, 24]; and passive EMAs for symptom monitoring [7, 13–15, 27].

Smart technologies (e.g., wearables or smartphone apps) were considered important for helping people recognise and understand their disorder [7, 13, 27], improving detection of the characteristics of unstable mood (including associated behaviour change and patterns of activity, texting, interaction with social media) [13, 14], overcoming limitations of self‐report and recall, and picking up signs of deterioration [7]. One participant cautioned that for some young people, monitoring can be an unwanted reminder of BD and EMAs are not a therapy substitute [15]. Another participant mentioned that there are over 10,000 mental health apps and although some appear usable and feasible, most are untested [7]. Digital interventions were seen as having dosing and engagement problems [9, 15] and requiring clinician‐supported access, navigation, and engagement [24]. Testing and validation of technologies was considered essential [13, 15, 17].

Two participants viewed the BD field as neglectful of new treatment discovery [5, 7]. A range of therapies was viewed as underused [13, 27], including ketogenic diet [27], transcranial magnetic stimulation (TMS) [13], bright light therapy [13], and pharmacotherapies that act on melatonin [13].

Theme 7 *Service Capacity and Models*. “*There is a lack of capacity and skill among clinicians across a broad range of services to diagnose and treat early‐stage BD. It is unclear which service model is best equipped to support the early stages of BD*”.

Participants agreed that primary care settings are where young people with BD are likely to be present but are also likely to be unrecognised and not appropriately treated [2, 8, 9, 16]. Similarly, participants reported that BD is not a priority in mental health services and is likely to be overlooked [5, 18, 20]. A contributing factor is that clinicians in these settings see too few patients with the disorder and as such have little experience, knowledge, and confidence in diagnosis and treatment [18]. Further, participants reported that individuals with BD are commonly faced with service barriers including access delays [11, 19], high thresholds for service entry [8, 11, 19, 24], inadequate follow‐up after diagnosis or hospital discharge [19, 20], and lack of continuity of care [5, 20].

Opinions were mixed regarding whether there should be specialist early intervention services for BD, or whether treatment should be delivered in complex mood disorders or transdiagnostic youth mental health services. Specialised clinical expertise in BD was viewed as hard to achieve in the absence of specialised BD services because clinicians do not see enough people with BD [18]. Some participants suggested it may be best to focus early intervention for BD in EIP services [8, 9, 14]. Others suggested that EIP is likely to be unhelpful as BD requires treatment by clinicians with expertise in mood disorders [8, 9]. Another concern was that EIP services are limited to people with psychotic symptoms; many with BD do not present with psychosis [9, 11]. One participant suggested that for EIP services to extend to BD, a culture change was required around the possibility of BD in clients [14].

Theme 8 *Families*. “*Families have a key role in assessment and treatment, and should be better supported*”.

Families were viewed as an underutilised and valuable resource in detecting early warning signs, diagnosis and treatment [3, 7, 10, 11, 13, 15, 16, 19, 24–27]. Participants expressed concern that collateral information from family members was often not taken seriously, and families must advocate for consideration of family history, possible BD diagnosis, and appropriate treatment [3, 9, 16, 19].

Families were also reported to need better support [3, 7, 10–13, 19, 24, 25]. One participant suggested that families are poorly supported even during acute episodes, but this was the ideal time for family peer support [10]. In families with a history of BD, education regarding early signs of BD could be helpful to aid early detection [3, 25]. Family peer and professional support groups for parents of young people at risk or in the early stages of BD could assist with recognition of BD but are not available [27]. FFT was recognised as a useful treatment for early intervention in BD [4, 9, 10, 14–16, 19–21, 25].

Theme 9 *Collaboration*. “*To advance early intervention in BD, we need to look at new opportunities to collaborate that involve all stakeholders, including those with a lived experience of BD*”.

Most participants advocated for improved collaborations [1, 6–10, 12–15, 17–19, 21–26]. Lack of collaboration was reported across stakeholder groups, including the interface between researchers, clinicians, service providers, and funding bodies [1, 9, 15, 17, 19]. There was recognition that people with lived experience and families should be more involved in collaborative work with stakeholders, including researchers [11, 13, 19, 26].

## 4 Discussion

Despite BD being a leading cause of disability, early intervention efforts for the disorder are in their infancy and there is much work to be done. Here we have taken a novel approach by consulting with international experts and using rigorous qualitative methodologies to identify barriers and challenges to advancing early intervention for BD.

### 4.1 Promoting Awareness

Although community awareness of BD has increased [33], especially driven by media and celebrities reporting a lived experience [34], experts report that awareness is not where it should be; this contributes to diagnostic delay and stigma [35]. Literacy of BD in the community is lower than for schizophrenia, ADHD, and autism [36]. Community awareness campaigns can improve awareness, but they need to be informed by a stronger evidence‐base of early warning signs and prodromal symptoms [35]. Having an international campaign would be helpful; similar approaches have been successfully implemented for psychosis [37].

Medical physicians and other clinicians also have poor literacy regarding at‐risk states, diagnosis, and treatment. Because of time restrictions, depression often being the incipient episode, lack of clarity around prodrome, the presence of comorbidities, and lack of screening and monitoring tools, BD is likely to go unrecognised [38, 39]. A recent study showed 30% of people receiving psychological treatment for depression in the UK's primary care therapy initiative have undiagnosed BD [40]. Another study estimated that three in 20 patients in general practice have undiagnosed bipolar depression [41]. If clinicians could have improved training and resources, then this might aid with earlier detection of the disorder, better coordination of treatments and referrals to secondary and/or tertiary care when needed, and help improve the management of physical health comorbidities (e.g., metabolic syndrome) [41].

### 4.2 Better Definitions, Measurement, and Data

Definitional problems plague BD, but consensus is essential for accurate identification of at‐risk states, diagnosis, and delineation of the course and outcomes of BD [42]. For research, consensus is important for allowing meaningful comparison across studies, especially around population identification, selection of measurement and outcome tools, data harmonisation, and strengthening the evidence‐base around treatment response and predictors of poor outcome [42]. There have been attempts to drive consensus definitions through the International Society of Bipolar Disorders (ISBD) and specific taskforces focusing on nomenclature of course and outcomes [42], clinical staging [43], precursors and prodromes [44], and cognition [45]; but validation of these definitions is needed [42].

Related to definitional problems are issues with measurement. Research on the psychometrics of tools to assess BD lags behind other disorders [46]. Most tools have been designed for adult populations [46, 47], a problem facing psychiatric research more broadly [48]. There is little focus on young people at‐risk of, or experiencing, the early stages of BD; this is surprising given that the typical age of onset of BD is between 15 and 24 years [45]. Often the focus is on manic symptom severity and diagnostic interviews, but BD is multifaceted and there is a need to consider other symptoms (atypical depression, hypomania) [46], social functioning [46], sleep and circadian rhythms [49, 50], cognition [45, 51], and quality of life [46]. More precise tools are needed to detect early warning signs [52], for BD II (including hypomanic symptoms) [46], as well as for comorbidities [53]. Screening tools are needed for community samples and there needs to be better patient‐reported measures [46]. There is also a growing interest in using technology for assessment purposes [54, 55, 56]. Furthermore, input from those with a lived experience when developing these definitions and measurement tools, is often lacking but essential for ensuring relevance to those affected by BD [42]. Whether using pre‐existing or new measures, there needs to be reasonable consensus about the best measurement tools to be implemented in research and clinical settings. In research settings, harmonisation of tools would allow greater comparability of research data across studies, bigger datasets, and can allow application of newer machine learning techniques to better understand the complexity of BD [57]. There also needs to be allowance for innovation in measurement and new tools to be validated in large cohorts.

### 4.3 Treatment Support and Innovation

The evidence base for high‐quality pharmacological, psychological, and service‐based interventions for the early stages of BD is scarce [24, 25, 27], an issue compounded by the lack of diagnostic specificity of early warning signs indicating vulnerability to the disorder [29]. Clinical guidelines provide inconsistent recommendations regarding treatments, particularly around the first episode of mania, maintenance treatments, and how to manage comorbidities [25, 27]. However, clinical guidelines need to be informed by high‐quality trials and real‐world data that not only assess the impact of treatments on symptoms but also on other factors such as tolerability and functioning [25].

Although lithium is the first‐line treatment for the disorder (especially for maintenance therapy and prophylaxis), prescription rates have declined by ~50%, which in part relates to clinicians' low levels of confidence and knowledge around the medication including dosage, plasma monitoring, and term side effects [58, 59, 60]. There is some evidence that lithium can have positive effects in young people, including improved symptomatic and functional recovery [61], reduced recurrence of mood episodes [25, 61] and suicide attempts [62], improved cognition [63], and reduced white matter alterations [64]. More evidence is required from larger trials comparing the effectiveness and tolerability of lithium compared to other mood stabilisers and/or antipsychotics, as well as an adjunct to psychosocial interventions [25]. The effectiveness and tolerability of lithium in at‐risk populations also requires consideration of the ethics of treating an individual who may not develop BD [24].

We are still unclear which treatments, or treatment combinations, work best for individuals at different disorder stages. Although clinical trials are the gold standard for testing the effectiveness and tolerability of interventions, generalisability to clinical populations can be limited and implementation into clinical practice slow [65]. We need to consider innovative ways to ensure effective treatments are translated into practice much earlier. This can extend to what service models best support individuals with BD, such as innovative specialised BD‐specific service models [66] or blended care models [67].

### 4.4 Limitations

This study has several limitations. The participation rate was 54.9%, which might reflect the tight recruitment timelines; a longer period could have yielded a higher rate. Nonetheless, the distribution of expert types and geographic locations between participants and non‐participants was comparable, and additional recruitment would likely not have altered the findings. Data saturation was achieved, with no new themes or insights emerging from the interviews, suggesting the findings were robust [68, 69].

We reported only limited demographic information about the cohort. Given the relatively small field of early intervention in BD, greater detail could have risked compromising participant confidentiality. Although we included a diverse range of perspectives across expert types and geographic regions, subgroup analyses were not possible due to small numbers and the need to protect identities. Finally, perspectives from experts in low‐ and middle‐income countries were not captured, and thus our findings may not fully represent global views on early intervention for BD.

## 5 Conclusions

In this study, we interviewed experts on advancing early intervention for BD, a field still in its infancy. There is a consensus on the need for clinical, research, and advocacy work. The engagement of participants in this study was encouraging with a shared goal to improve early intervention for BD. Developing collaborative networks that include individuals with a lived experience is vital for advocacy and research funding, potentially transforming early intervention and improving outcomes for those impacted by the disorder. There is much work to be done.

## Funding

The Daymark Foundation provided financial support to SC and MH at Orygen for the conduct of this research.

## Conflicts of Interest

The authors declare no conflicts of interest.

## Data Availability

The authors have nothing to report.

## References

[1] J. Scott , A. Graham , A. Yung , C. Morgan , F. Bellivier , and B. Etain , “A Systematic Review and Meta‐Analysis of Delayed Help‐Seeking, Delayed Diagnosis and Duration of Untreated Illness in Bipolar Disorders,” Acta Psychiatrica Scandinavica 146, no. 5 (2022): 389–405, 10.1111/acps.13490. 36018259

[2] J. S. Carpenter , J. Scott , F. Iorfino , et al., “Predicting the Emergence of Full‐Threshold Bipolar I, Bipolar II and Psychotic Disorders in Young People Presenting to Early Intervention Mental Health Services,” Psychological Medicine 52, no. 10 (2022): 1990–2000, 10.1017/s0033291720003840. 33121545

[3] H. Grunze and C. Born , “The Impact of Subsyndromal Bipolar Symptoms on Patient's Functionality and Quality of Life,” Frontiers in Psychiatry 11 (2020): 510, 10.3389/fpsyt.2020.00510. 32595531 PMC7304232

[4] S. Greenberg , K. L. Rosenblum , M. G. McInnis , and M. Muzik , “The Role of Social Relationships in Bipolar Disorder: A Review,” Psychiatry Research 219, no. 2 (2014): 248–254, 10.1016/j.psychres.2014.05.047. 24947918

[5] S. Marwaha , A. Durrani , and S. Singh , “Employment Outcomes in People With Bipolar Disorder: A Systematic Review,” Acta Psychiatrica Scandinavica 128, no. 3 (2013): 179–193, 10.1111/acps.12087. 23379960

[6] S. M. Cotton , K. M. Filia , M. Lambert , et al., “Not in Education, Employment and Training Status in the Early Stages of Bipolar I Disorder With Psychotic Features,” Early Intervention in Psychiatry 16, no. 6 (2022): 609–617, 10.1111/eip.13203. 34313390

[7] K. M. Filia , S. M. Cotton , A. E. Watson , A. Jayasinghe , M. Kerr , and P. B. Fitzgerald , “Understanding the Barriers and Facilitators to Employment for People With Bipolar Disorder,” Psychiatric Quarterly 92, no. 4 (2021): 1565–1579, 10.1007/s11126-021-09931-w. 34097245

[8] E. E. Michalak , L. N. Yatham , V. Maxwell , S. Hale , and R. W. Lam , “The Impact of Bipolar Disorder Upon Work Functioning: A Qualitative Analysis,” Bipolar Disorders 9, no. 1–2 (2007): 126–143, 10.1111/j.1399-5618.2007.00436.x. 17391356

[9] F. Goodwin and K. Jamison , Manic Depressive Illness (Oxford University Press, 1990).

[10] C. Miller and M. S. Bauer , “Excess Mortality in Bipolar Disorders,” Current Psychiatry Reports 16 (2014): 499. 25194314 10.1007/s11920-014-0499-z

[11] J. F. Hayes , J. Miles , K. Walters , M. King , and D. P. J. Osborn , “A Systematic Review and Meta‐Analysis of Premature Mortality in Bipolar Affective Disorder,” Acta Psychiatrica Scandinavica 131, no. 6 (2015): 417–425, 10.1111/acps.12408. 25735195 PMC4939858

[12] GBD 2019 Mental Disorders Collaborators , “Global, Regional, and National Burden of 12 Mental Disorders in 204 Countries and Territories. 1990–2019: A Systematic Analysis From the Global Burden of Disease Study 2019,” Lancet Psychiatry 336 (2020): 1223–1249. 10.1016/S2215-0366(21)00395-3 PMC8776563 35026139

[13] G. Scaini , S. S. Valvassori , A. P. Diaz , et al., “Neurobiology of Bipolar Disorders: A Review of Genetic Components, Signaling Pathways, Biochemical Changes, and Neuroimaging Findings,” Brazilian Journal of Psychiatry 42, no. 5 (2020): 536–551, 10.1590/1516-4446-2019-0732. 32267339 PMC7524405

[14] T. A. Rowland and S. Marwaha , “Epidemiology and Risk Factors for Bipolar Disorder,” Therapeutic Advances in Psychopharmacology 8 (2018): 251–269. 30181867 10.1177/2045125318769235 PMC6116765

[15] Á. Lublóy , J. L. Keresztúri , A. Németh , and P. Mihalicza , “Exploring Factors of Diagnostic Delay for Patients With Bipolar Disorder: A Population‐Based Cohort Study,” BMC Psychiatry 20, no. 1 (2020): 75, 10.1186/s12888-020-2483-y. 32075625 PMC7031950

[16] Z. Wang , J. Chen , C. Zhang , et al., “Guidelines Concordance of Maintenance Treatment in Euthymic Patients With Bipolar Disorder: Data From the National Bipolar Mania Pathway Survey (BIPAS) in Mainland China,” Journal of Affective Disorders 182 (2015): 101–105. 25983305 10.1016/j.jad.2015.04.028

[17] P. J. Harrison , A. Cipriani , C. J. Harmer , et al., “Innovative Approaches to Bipolar Disorder and Its Treatment,” Annals of the New York Academy of Sciences 1366, no. 1 (2016): 76–89, 10.1111/nyas.13048. 27111134 PMC4850752

[18] J. F. Cade , “Lithium Salts in the Treatment of Psychotic Excitement,” Medical Journal of Australia 2 (1949): 349–352. 18142718 10.1080/j.1440-1614.1999.06241.x

[19] J. H. Baek , K. Ha , L. N. Yatham , et al., “Pattern of Pharmacolotherapy by Episode Types for Patients With Bipolar Disorders and Its Concordance With Treatment Guidelines,” Journal of Clinical Psychopharmacology 34 (2014): 577–587. 25006813 10.1097/JCP.0000000000000175

[20] L. M. Weinstock , B. A. Gaudiano , G. Epstein‐Lubow , K. Tezanos , C. E. Celis‐deHoyos , and I. W. Miller , “Medication Burden in Bipolar Disorder: A Chart Review of Patients at Psychiatric Hospital Admission,” Psychiatry Research 216 (2014): 24–30. 24534121 10.1016/j.psychres.2014.01.038 PMC3968952

[21] G. Murray , N. D. Leitan , N. Thomas , et al., “Towards Recovery‐Oriented Psychosocial Interventions for Bipolar Disorder: Quality of Life Outcomes, Stage‐Sensitive Treatments, and Mindfulness Mechanisms,” Clinical Psychology Review 52 (2017): 148–163. 28129636 10.1016/j.cpr.2017.01.002

[22] M. Hajda , J. Prasko , K. Latalova , et al., “Unmet Needs of Bipolar Disorder Patients,” Neuropsychiatric Disease and Treatment 12 (2016): 1561–1570, 10.2147/NDT.S105728. 27445475 PMC4928671

[23] A. Barbato , M. Vallarino , F. Rapisarda , et al., “Do People With Bipolar Disorders Have Access to Psychosocial Treatments? A Survey in Italy,” International Journal of Social Psychiatry 62, no. 4 (2016): 334–344, 10.1177/0020764016631368. 26896028

[24] E. Vieta , E. Salagre , I. Grande , et al., “Early Intervention in Bipolar Disorder,” American Journal of Psychiatry 175, no. 5 (2018): 411–426, 10.1176/appi.ajp.2017.17090972. 29361850

[25] A. Ratheesh , D. Hett , J. Ramain , et al., “A Systematic Review of Interventions in the Early Course of Bipolar Disorder I or II: A Report of the International Society for Bipolar Disorders Taskforce on Early Intervention,” International Journal of Bipolar Disorders 11, no. 1 (2023): 1, 10.1186/s40345-022-00275-3. 36595095 PMC9810772

[26] X. Benarous , A. Consoli , V. Milhiet , and D. Cohen , “Early Interventions for Youths at High Risk for Bipolar Disorder: A Developmental Approach,” European Child & Adolescent Psychiatry 25, no. 3 (2016): 217–233, 10.1007/s00787-015-0773-6. 26395448

[27] S. Jauhar , A. Ratheesh , C. Davey , et al., “The Case for Improved Care and Provision of Treatment for People With First‐Episode Mania,” Lancet Psychiatry 6, no. 10 (2019): 869–876, 10.1016/s2215-0366(19)30082-3. 31248840

[28] A. J. Ferrari , E. Stockings , J. P. Khoo , et al., “The Prevalence and Burden of Bipolar Disorder: Findings Fromt the Global Burden of Disease Study 2013,” Bipolar Disorders 18 (2016): 440–450. 27566286 10.1111/bdi.12423

[29] M. Vallarino , C. Henry , B. Etain , et al., “An Evidence Map of Psychosocial Interventions for the Earliest Stages of Bipolar Disorder,” Lancet Psychiatry 2 (2015): 548–563. 26360451 10.1016/S2215-0366(15)00156-X PMC4629930

[30] P. D. McGorry , A. Ratheesh , and B. O'Donoghue , “Early Intervention‐An Implementation Challenge for 21st Century Mental Health Care,” JAMA Psychiatry 75, no. 6 (2018): 545–546, 10.1001/jamapsychiatry.2018.0621. 29801060

[31] B. C. O'Brien , I. B. Harris , T. J. Beckman , D. A. Reed , and D. A. Cook , “Standards for Reporting Qualitative Research: A Synthesis of Recommendations,” Academic Medicine 89, no. 9 (2014): 1245–1251. 24979285 10.1097/ACM.0000000000000388

[32] V. Braun and V. Clarke , Thematic Analysis: A Practical Guide (SAGE, 2022).

[33] M. C. Angermeyer , M. G. Carta , A. Holzinger , and H. Matschinger , “The Diffusion of the Diagnostic Term Bipolar Disorder Among the German Public,” Psychiatry Research 260 (2018): 75–77, 10.1016/j.psychres.2017.11.047. 29175502

[34] N. C. H. Wong , K. L. Lookadoo , and G. S. Nisbett , ““I'm Demi and I Have Bipolar Disorder”: Effect of Parasocial Contact on Reducing Stigma Toward People With Bipolar Disorder,” Communication Studies 68, no. 3 (2017): 314–333, 10.1080/10510974.2017.1331928.

[35] G. Perna , T. Torti , and A. Alciati , “Effects of Public Awareness and Stigmatization on Accurate and Timely Diagnosis of Bipolar Disorder,” in The Bipolar Book: History, Neurobiology, and Treatment, ed. A. Yildiz and C. Nemeroff (Oxford University Press, 2015).

[36] F. Vovou , L. Hull , and K. V. Petrides , “Mental Health Literacy of ADHD, Autism, Schizophrenia, and Bipolar Disorder: A Cross‐Cultural Investigation,” Journal of Mental Health 30, no. 4 (2021): 470–480, 10.1080/09638237.2020.1713999. 31994950

[37] B. Lloyd‐Evans , M. Crosby , S. Stockton , et al., “Initiatives to Shorten Duration of Untreated Psychosis: Systematic Review,” British Journal of Psychiatry 198, no. 4 (2011): 256–263, 10.1192/bjp.bp.109.075622. 21972275

[38] M. E. Thase , S. M. Stahl , R. S. McIntyre , et al., “Screening for Bipolar I Disorder and the Rapid Mood Screener: Results of a Nationwide Health Care Provider Survey,” Primary Care Companion CNS Disorders 25, no. 2 (2023): 22m03322, 10.4088/PCC.22m03322. 37115145

[39] C. Schiweck , G. Arteaga‐Henriquez , M. Aichholzer , et al., “Comorbidity of ADHD and Adult Bipolar Disorder: A Systematic Review and Meta‐Analysis,” Neuroscience and Biobehavioral Reviews 124 (2021): 100–123, 10.1016/j.neubiorev.2021.01.017. 33515607

[40] R. Strawbridge , L. Alexander , T. Richardson , A. H. Young , and A. J. Cleare , “Is There a ‘Bipolar Iceberg’ in UK Primary Care Psychological Therapy Services?,” Psychological Medicine 53, no. 12 (2023): 5385–5394, 10.1017/s0033291722002343. 35920607 PMC10482719

[41] H. Huang , N. Nissen , C. T. Lim , J. L. Gören , M. Spottswood , and H. Huang , “Treating Bipolar Disorder in Primary Care: Diagnosis, Pharmacology, and Management,” International Journal of General Medicine 15 (2022): 8299–8314, 10.2147/ijgm.S386875. 36447648 PMC9701507

[42] M. Tohen , E. Frank , C. L. Bowden , et al., “The International Society for Bipolar Disorders (ISBD) Task Force Report on the Nomenclature of Course and Outcome in Bipolar Disorders,” Bipolar Disorders 11, no. 5 (2009): 453–473, 10.1111/j.1399-5618.2009.00726.x. 19624385

[43] R. Kupka , A. Duffy , J. Scott , et al., “Consensus on Nomenclature for Clinical Staging Models in Bipolar Disorder: A Narrative Review From the International Society for Bipolar Disorders (ISBD) Staging Task Force,” Bipolar Disorders 23 (2021): 659–678, 10.1111/bdi.13105. 34174130 PMC9290926

[44] G. L. Faedda , R. J. Baldessarini , C. Marangoni , et al., “An International Society of Bipolar Disorders Task Force Report: Precursors and Prodromes of Bipolar Disorder,” Bipolar Disorders 21, no. 8 (2019): 720–740, 10.1111/bdi.12831. 31479581

[45] K. Miskowiak , K. Burdick , A. Martinez‐Aran , et al., “Assessing and Addressing Cognitive Impairment in Bipolar Disorder: The International Society for Bipolar Disorders Targeting Cognition Task Force Recommendations for Clinicians,” Bipolar Disorders 20, no. 3 (2018): 184–194, 10.1111/bdi.12595. 29345040

[46] C. J. Miller , S. L. Johnson , and L. Eisner , “Assessment Tools for Adult Bipolar Disorder,” Clinical Psychology (New York) 16, no. 2 (2009): 188–201, 10.1111/j.1468-2850.2009.01158.x. PMC2847794 20360999

[47] T. D. Meyer , N. Crist , N. La Rosa , B. Ye , J. C. Soares , and I. E. Bauer , “Are Existing Self‐Ratings of Acute Manic Symptoms in Adults Reliable and Valid?‐A Systematic Review,” Bipolar Disorders 22, no. 6 (2020): 558–568, 10.1111/bdi.12906. 32232950

[48] S. M. Cotton , K. M. Filia , M. P. Hamilton , et al., “Accelerating Youth Mental Health Services Research,” Australasian Psychiatry 31 (2023): 292–294, 10.1177/10398562231167691. 37021582

[49] E. Morton and G. Murray , “Assessment and Treatment of Sleep Problems in Bipolar Disorder‐A Guide for Psychologists and Clinically Focused Review,” Clinical Psychology & Psychotherapy 27, no. 3 (2020): 364–377, 10.1002/cpp.2433. 32068325

[50] I. B. Hickie , K. R. Merikangas , J. S. Carpenter , et al., “Does Circadian Dysrhythmia Drive the Switch Into High‐ or Low‐Activation States in Bipolar I Disorder?,” Bipolar Disorders 25, no. 3 (2023): 191–199, 10.1111/bdi.13304. 36661342 PMC10947388

[51] M. G. Rossetti , F. Girelli , C. Perlini , P. Brambilla , and M. Bellani , “Neuropsychological Instruments for Bipolar Disorders: A Systematic Review on Psychometric Properties,” Journal of Affective Disorders 338 (2023): 358–364, 10.1016/j.jad.2023.06.026. 37331381

[52] C. F. Baldassano , “Assessment Tools for Screening and Monitoring Bipolar Disorder,” Bipolar Disorders 7, no. 1 (2005): 8–15, 10.1111/j.1399-5618.2005.00189.x. 15762864

[53] J. F. Goldberg , “Comorbidity in Bipolar Disorder: Assessment and Treatment,” in Bipolar Disorder: A Clinician's Guide to Treatment Managment, ed. L. Yatham and V. Kusumaker (Taylor and Francis Group, 2009).

[54] E. C. Chan , Y. Sun , K. J. Aitchison , and S. Sivapalan , “Mobile App‐Based Self‐Report Questionnaires for the Assessment and Monitoring of Bipolar Disorder: Systematic Review,” JMIR Formative Research 5, no. 1 (2021): e13770, 10.2196/13770. 33416510 PMC7822726

[55] H. Li , D. Mukherjee , V. B. Krishnamurthy , et al., “Use of Ecological Momentary Assessment to Detect Variability in Mood, Sleep and Stress in Bipolar Disorder,” BMC Research Notes 12, no. 1 (2019): 791, 10.1186/s13104-019-4834-7. 31801608 PMC6894147

[56] A. Rajagopalan , P. Shah , M. W. Zhang , and R. C. Ho , “Digital Platforms in the Assessment and Monitoring of Patients With Bipolar Disorder,” Brain Sciences 7, no. 11 (2017): 150, 10.3390/brainsci7110150. 29137156 PMC5704157

[57] M. Manchia , E. Vieta , O. B. Smeland , et al., “Translating Big Data to Better Treatment in Bipolar Disorder ‐ a Manifesto for Coordinated Action,” European Neuropsychopharmacology 36 (2020): 121–136, 10.1016/j.euroneuro.2020.05.006. 32536571

[58] G. S. Malhi , E. Bell , M. Jadidi , M. Gitlin , and M. Bauer , “Countering the Declining Use of Lithium Therapy: A Call to Arms,” International Journal of Bipolar Disorders 11, no. 1 (2023): 30, 10.1186/s40345-023-00310-x. 37633877 PMC10460327

[59] Y. K. Shuy , S. Santharan , Q. H. Chew , and K. Sim , “International Trends in Lithium Use for Pharmacotherapy and Clinical Correlates in Bipolar Disorder: A Scoping Review,” Brain Sciences 14, no. 1 (2024): 102, 10.3390/brainsci14010102. 38275522 PMC10813799

[60] L. V. Kessing , “Why Is Lithium [Not] the Drug of Choice for Bipolar Disorder? A Controversy Between Science and Clinical Practice,” International Journal of Bipolar Disorders 12, no. 1 (2024): 3, 10.1186/s40345-023-00322-7. 38228882 PMC10792154

[61] M. Berk , R. Daglas , O. Dandash , et al., “Quetiapine v. Lithium in the Maintenance Phase Following a First Episode of Mania: Randomised Controlled Trial,” British Journal of Psychiatry 210 (2017): 413–421. 10.1192/bjp.bp.116.186833 28254958

[62] D. M. Hafeman , B. Rooks , J. Merranko , et al., “Lithium Versus Other Mood‐Stabilizing Medications in a Longitudinal Study of Youth Diagnosed With Bipolar Disorder,” Journal of the American Academy of Child and Adolescent Psychiatry 59, no. 10 (2020): 1146–1155, 10.1016/j.jaac.2019.06.013. 31369795 PMC6987013

[63] R. Daglas , S. M. Cotton , K. Allott , et al., “A Single‐Blind, Randomised Controlled Trial on the Effects of Lithium and Quetiapine Monotherapy on the Trajectory of Cognitive Functioning in First Episode Mania: A 12‐Month Follow‐Up Study,” European Psychiatry 31 (2016): 20–28. 26655594 10.1016/j.eurpsy.2015.09.460

[64] M. Berk , O. Dandash , R. Daglas , et al., “Neuroprotection After a First Episode of Mania: A Randomized Controlled Maintenance Trial to Assess the Effects of Lithium Compared to Quetiapine on Regional Grey and White Matter Volume,” Translational Psychiatry 7 (2017): e1041. 28221364 10.1038/tp.2017.13 PMC5438029

[65] Z. S. Morris , S. Wooding , and J. Grant , “The Answer Is 17 Years, What Is the Question: Understanding Time Lags in Translational Research,” Journal of the Royal Society of Medicine 104, no. 12 (2011): 510–520, 10.1258/jrsm.2011.110180. 22179294 PMC3241518

[66] K. Miley , P. Meyer‐Kalos , C. Coudray , et al., “Expanding Coordinated Specialty Care to Early‐Stagebipolar Disorder: Initial Implementation and Feasibility of the Strideintervention,” Bipolar Disorders 126 (2024): 128.

[67] A. Ratheesh , J. Gates , D. Hammond , et al., “Bipolar Early Intervention Using New Digital Technologies (BLEND): A Pilot Randomised Controlled Trial of a Novel Blended‐Digital Early Intervention Model of Care for Youth With Bipolar Disorder I or II,” Early Intervention in Psychiatry 19, no. 6 (2025): e70060, 10.1111/eip.70060. 40539376 PMC12179817

[68] B. Saunders , J. Sim , T. Kingstone , et al., “Saturation in Qualitative Research: Exploring Its Conceptualization and Operationalization,” Quality & Quantity 52, no. 4 (2018): 1893–1907, 10.1007/s11135-017-0574-8. 29937585 PMC5993836

[69] S. K. Ahmed , “Sample Size for Saturation in Qualitative Research: Debates, Definitions, and Strategies,” Journal of Medicine, Surgery and Public Health 5 (2025): 100171, 10.1016/j.glmedi.2024.100171.

